# A Good Investment of Money

**Published:** 1889-03

**Authors:** 


### A GOOD INVESTMENT OF MONEY.

The advantages of attending society meetings are clearly and forcibly pointed out in an editorial in the American Lancet for January.

We said in our last issue that the Texas State Medical Association should make its meetings a necessity in order to attract its members. We should have said also, that a more thorough appreciation on the part of practitioners of medicine of the many and great advantages to be derived from a regular attendance upon, and participation in the meetings, should be inculcated. Were this fully understood there would be no complaint of scant attendance.

We would be glad to reproduce the Lancet's article if we had room, in the hope that it might aid us in inducing physicians to come to San Antonio next month. Those members who attend regularly the meetings of the Texas State Medical Association become extensively known, personally, not only in their own State, but abroad; for they frequently meet there, distinguished visitors from sister societies, and the representatives of the large manufacturing houses, north and east; and meet also brother practitioners from all parts of the State, between whom and themselves warm friendship not infrequently springs up. We can point out not a few, who, a short time ago, were considered obscure, and not above mediocrity, who have become foremost in the profession; not by merely attending the meetings, but because they have, by debate, or by written contributions, developed their thoughts, and made them known; have shown what was in them, which, but for opportunity, would have remained "under a bushel," all their days. There can be no doubt that the intellectual powers are sharpened by the discussion of medical topics, orally, or in writing; by the interchange of ideas the mind is developed, and one's views and opinions are broadened, and he is improved in many ways. The practice too, of discussion, gives one confidence in himself, and he soon becomes familiar with, and used to off-hand speaking before a crowd. This is a great advantage ; for, as the Lancet points out, medical men are more largely estimated by the public than they are aware of, by their ability, or, lack of it, to discuss medical or sanitary topics intelligently, and to express their views clearly and forcibly. One who can do this is considered master of his profession. We all know that there are many profound thinkers and accomplished physicians in our ranks who cannot express themselves clearly in a conversation, and who would appear to great disadvantage if called on for an opinion before court, or in a meeting of citizens. The practice afforded in debate in our societies, goes a long way toward developing and polishing one's gifts in this regard.

Besides the above, and the points suggested by the Lancet, the attendants upon the meetings of their medical societies, keep fully posted in medical and sanitary progress, as well as in medical politics; they hear for the first time at a society meeting, of some new inventions or discovery; or get a good idea from the experience of a brother practitioner, which may help them sometime, in an emergency.

The money cost of four days attendance on the State Association Convention is insignificant, compared with the many and great benefits derived. We hope to see a full representation on the 23d prox. So be it.

---

PROF. RICHARDSON, of New Orleans, is in such bad health as to be comdelled to relinquish for a while the Chair of Emeritus Professor of Anatomy in Tulane University.

